# *rac*-Diethyl 6-hy­droxy-4-[(2-hy­droxy­eth­yl)amino]-6-methyl-2-phenyl­cyclo­hex-3-ene-1,3-dicarboxyl­ate

**DOI:** 10.1107/S1600536810052669

**Published:** 2010-12-18

**Authors:** Abel M. Maharramov, Arif I. Ismiyev, Bahruz A. Rashidov

**Affiliations:** a Baku State University, Z. Khalilov St. 23, Baku, AZ-1148, Azerbaijan

## Abstract

The title compound, C_21_H_29_NO_6_, is chiral with three stereogenic centres. The crystal is a racemate and consists of enanti­omeric pairs with the relative configuration *rac*-(2R*,3S*,4R*). The ethyl fragment of the eth­oxy­carbonyl group at position 1 is disordered in a 0.60:0.40 ratio. The crystal packing displays inter­molecular O—H⋯O hydrogen bonding. An intra­molecular N—H⋯O hydrogen bond also occurs.

## Related literature

β-Cyclo­ketoles and their nitro­genous derivatives possess a wide spectrum of biological activity, see: Krivenko *et al.* (2003 ▶).
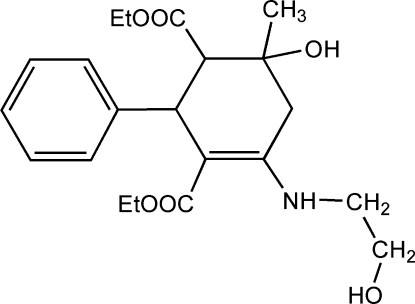


## Experimental

### 

#### Crystal data

C_21_H_29_NO_6_
*M* *_r_* = 391.45
Monoclinic, 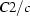

*a* = 41.078 (14) Å
*b* = 5.940 (2) Å
*c* = 18.683 (6) Å
β = 113.581 (13)°
*V* = 4178 (2) Å^3^
*Z* = 8
Mo *K*α radiation
μ = 0.09 mm^−1^
*T* = 100 K
0.30 × 0.30 × 0.30 mm

#### Data collection

Bruker APEXII CCD diffractometer
Absorption correction: multi-scan (*SADABS*; Sheldrick, 1998 ▶) *T*_min_ = 0.973, *T*_max_ = 0.973
18275 measured reflections
4530 independent reflections
3052 reflections with *I* > 2σ(*I*)
*R*_int_ = 0.060

#### Refinement

*R*[*F* ^2^ > 2σ(*F* ^2^)] = 0.052
*wR*(*F* ^2^) = 0.143
*S* = 1.00
4530 reflections
263 parameters
8 restraints
H-atom parameters constrained
Δρ_max_ = 0.27 e Å^−3^
Δρ_min_ = −0.25 e Å^−3^

### 

Data collection: *APEX2* (Bruker, 2005 ▶); cell refinement: *SAINT-Plus* (Bruker, 2001 ▶); data reduction: *SAINT-Plus*; program(s) used to solve structure: *SHELXTL* (Sheldrick, 2008 ▶); program(s) used to refine structure: *SHELXTL*; molecular graphics: *SHELXTL*; software used to prepare material for publication: *SHELXTL*.

## Supplementary Material

Crystal structure: contains datablocks global, I. DOI: 10.1107/S1600536810052669/kp2299sup1.cif

Structure factors: contains datablocks I. DOI: 10.1107/S1600536810052669/kp2299Isup2.hkl

Additional supplementary materials:  crystallographic information; 3D view; checkCIF report

## Figures and Tables

**Table 1:** Hydrogen-bond geometry (Å, °)

*D*—H⋯*A*	*D*—H	H⋯*A*	*D*⋯*A*	*D*—H⋯*A*
O1—H1*O*⋯O6^i^	0.94	1.92	2.802 (2)	157
O6—H6*O*⋯O1^ii^	0.95	1.78	2.727 (2)	172
N1—H1*N*⋯O2	0.91	1.91	2.650 (2)	137

Symmetry codes: (i) 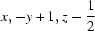; (ii) 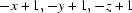.

**Fig. 1.**
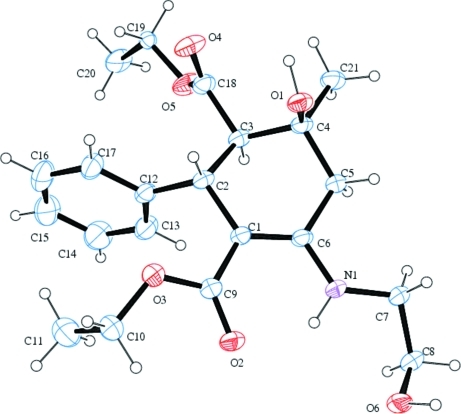
The molecular structure of the title compound with the atomic numbering scheme. Displacement ellipsoids were drawn at the 30% probability level.

**Fig. 2.**
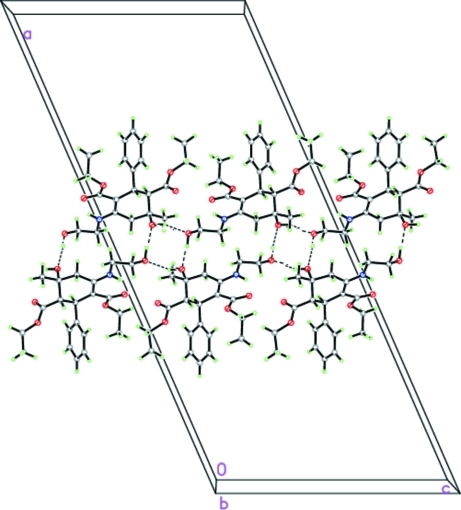
Crystal packing of the title compound with hydrogen bonds (dashed lines). H atoms not involved in hydrogen bonding have been omitted for clarity.

## supplementary crystallographic information

### Comment

β-Cycloketoles and their nitrogenous derivatives possess a wide spectrum of biological activity (Krivenko *et al.* 2003). The reactions of β-cycloketoles with ethanolamine possibly lead to valuable compounds of practical use but remain unexplored. Reaction β-cycloketoles with ethanolamine has not been studied. Several reaction paths may be expected: one or two reactive centres of the substrate and reagent may be involved. Enamines or the products of heterocyclisation or spirocyclisation may be produced.

The cyclohexene ring has a distorted half-chair conformation. Phenyl ring is in a pseudo-equatorial position (Fig. 1). Torsion angle between the ethoxycarbonyl group and the phenyl substituent, C12—C2—C3—C18 is 59,04 (18) °, indicating the pseudo-axial location of hydrogen atoms at C2 and C3. The crystal structure involves O—H···O intermolecular and O—H···O and N—H···O intramolecular hydrogen bonds (Table 1 and Fig. 2).

### Experimental

(rac)-Diethyl-4-hydroxy-4-methyl-6-oxo-2-phenyl-1,3-dicarboxylate (20 mmol), monoethanolamine (20 mmol) were dissolved in 20 mL ethanol. The mixture was stirred at 345–350 K within 10 h. After cooling to a room temperature white crystals were obtained. The crystals were filtered and washed with ethanol. Then the crystals obtained were recrystallised from 50 mL ethanol to yield colourless block-shaped crystals of the title compound.

### Refinement

The hydrogen atoms of the NH and OH-groups of (I) were localized in the difference-Fourier map and included in the refinement with fixed positional and isotropic displacement parameters [*U*_iso_(H) = 1.5*U*_eq_(C) for CH_3_-group and *U*_iso_(H) = 1.2*U*_eq_(N) for amino groups]. The other hydrogen atoms were placed in calculated positions with and refined in the riding model with fixed isotropic displacement parameters [*U*_iso_(H) = 1.2*U*_eq_(C)].

### Crystal data

**Table:**

C_21_H_29_NO_6_	*F*(000) = 1680
*M**_r_* = 391.45	*D*_x_ = 1.245 Mg m^−^^3^
Monoclinic, *C*2/*c*	Mo *K*α radiation, λ = 0.71073 Å
Hall symbol: -C 2yc	Cell parameters from 2594 reflections
*a* = 41.078 (14) Å	θ = 2.4–30.1°
*b* = 5.940 (2) Å	µ = 0.09 mm^−^^1^
*c* = 18.683 (6) Å	*T* = 100 K
β = 113.581 (13)°	Prism, colourless
*V* = 4178 (2) Å^3^	0.30 × 0.30 × 0.30 mm
*Z* = 8	

### Data collection

**Table:**

Bruker APEXII CCD diffractometer	4530 independent reflections
Radiation source: fine-focus sealed tube	3052 reflections with *I* > 2σ(*I*)
graphite	*R*_int_ = 0.060
φ and ω scans	θ_max_ = 27.0°, θ_min_ = 2.2°
Absorption correction: multi-scan (*SADABS*; Sheldrick, 1998)	*h* = −52→48
*T*_min_ = 0.973, *T*_max_ = 0.973	*k* = −7→7
18275 measured reflections	*l* = −23→23

### Refinement

**Table:**

Refinement on *F*^2^	Primary atom site location: structure-invariant direct methods
Least-squares matrix: full	Secondary atom site location: difference Fourier map
*R*[*F*^2^ > 2σ(*F*^2^)] = 0.052	Hydrogen site location: difference Fourier map
*wR*(*F*^2^) = 0.143	H-atom parameters constrained
*S* = 1.00	*w* = 1/[σ^2^(*F*_o_^2^) + (0.070*P*)^2^ + 1.6*P*] where *P* = (*F*_o_^2^ + 2*F*_c_^2^)/3
4530 reflections	(Δ/σ)_max_ < 0.001
263 parameters	Δρ_max_ = 0.27 e Å^−^^3^
8 restraints	Δρ_min_ = −0.25 e Å^−^^3^

### Special details

**Table:**

Geometry. All e.s.d.'s (except the e.s.d. in the dihedral angle between two l.s. planes) are estimated using the full covariance matrix. The cell e.s.d.'s are taken into account individually in the estimation of e.s.d.'s in distances, angles and torsion angles; correlations between e.s.d.'s in cell parameters are only used when they are defined by crystal symmetry. An approximate (isotropic) treatment of cell e.s.d.'s is used for estimating e.s.d.'s involving l.s. planes.
Refinement. Refinement of *F*^2^ against ALL reflections. The weighted *R*-factor *wR* and goodness of fit *S* are based on *F*^2^, conventional *R*-factors *R* are based on *F*, with *F* set to zero for negative *F*^2^. The threshold expression of *F*^2^ > σ(*F*^2^) is used only for calculating *R*-factors(gt) *etc*. and is not relevant to the choice of reflections for refinement. *R*-factors based on *F*^2^ are statistically about twice as large as those based on *F*, and *R*- factors based on ALL data will be even larger.

### Fractional atomic coordinates and isotropic or equivalent isotropic displacement parameters (Å^2^)

**Table:**

	*x*	*y*	*z*	*U*_iso_*/*U*_eq_	Occ. (<1)
O1	0.45627 (3)	0.6020 (2)	0.27020 (7)	0.0437 (3)	
H1O	0.4558	0.6389	0.2211	0.065*	
O2	0.40537 (4)	0.7311 (3)	0.49029 (7)	0.0610 (4)	
O3	0.38193 (4)	0.9619 (2)	0.38680 (7)	0.0524 (4)	
O4	0.39044 (4)	0.6792 (3)	0.11937 (8)	0.0648 (4)	
O5	0.35176 (4)	0.4026 (3)	0.10896 (7)	0.0627 (4)	
O6	0.47333 (4)	0.3324 (3)	0.64070 (7)	0.0546 (4)	
H6O	0.4984	0.3482	0.6684	0.082*	
N1	0.44548 (4)	0.3995 (3)	0.47590 (8)	0.0457 (4)	
H1N	0.4359	0.4776	0.5046	0.055*	
C1	0.40713 (5)	0.6258 (3)	0.36860 (9)	0.0373 (4)	
C2	0.38927 (5)	0.6718 (3)	0.28057 (9)	0.0375 (4)	
H2	0.4005	0.8089	0.2690	0.045*	
C3	0.39557 (5)	0.4713 (3)	0.23451 (9)	0.0385 (4)	
H3	0.3822	0.3388	0.2417	0.046*	
C4	0.43481 (5)	0.4067 (3)	0.26514 (9)	0.0390 (4)	
C5	0.44550 (5)	0.3190 (3)	0.34878 (9)	0.0399 (4)	
H5A	0.4369	0.1623	0.3461	0.048*	
H5B	0.4717	0.3150	0.3747	0.048*	
C6	0.43160 (5)	0.4552 (3)	0.39896 (9)	0.0368 (4)	
C7	0.47043 (6)	0.2210 (3)	0.51284 (10)	0.0471 (5)	
H7A	0.4949	0.2737	0.5246	0.056*	
H7B	0.4655	0.0905	0.4771	0.056*	
C8	0.46723 (6)	0.1506 (4)	0.58791 (10)	0.0506 (5)	
H8A	0.4432	0.0892	0.5753	0.061*	
H8B	0.4846	0.0295	0.6132	0.061*	
C9	0.39840 (5)	0.7687 (3)	0.42100 (10)	0.0424 (4)	
C10	0.36501 (6)	1.0945 (4)	0.42722 (13)	0.0575 (6)	
H10A	0.3704	1.2561	0.4246	0.069*	
H10B	0.3742	1.0496	0.4828	0.069*	
C11	0.32549 (7)	1.0567 (5)	0.38929 (16)	0.0751 (7)	
H11A	0.3138	1.1501	0.4152	0.113*	
H11B	0.3203	0.8977	0.3940	0.113*	
H11C	0.3167	1.0977	0.3339	0.113*	
C12	0.34941 (5)	0.7105 (3)	0.24955 (10)	0.0426 (4)	
C13	0.32735 (6)	0.5590 (4)	0.26467 (13)	0.0601 (6)	
H13	0.3375	0.4344	0.2981	0.072*	
C14	0.29093 (7)	0.5856 (5)	0.23212 (17)	0.0806 (8)	
H14	0.2763	0.4797	0.2433	0.097*	
C15	0.27578 (7)	0.7649 (6)	0.18358 (16)	0.0840 (8)	
H15	0.2507	0.7830	0.1611	0.101*	
C16	0.29710 (7)	0.9171 (5)	0.16787 (15)	0.0774 (8)	
H16	0.2868	1.0420	0.1348	0.093*	
C17	0.33352 (6)	0.8901 (4)	0.19988 (12)	0.0561 (5)	
H17	0.3480	0.9955	0.1878	0.067*	
C18	0.38008 (5)	0.5315 (3)	0.14896 (9)	0.0448 (4)	
C19	0.32971 (18)	0.4777 (12)	0.0292 (2)	0.0852 (16)	0.60
H19A	0.3422	0.4503	−0.0057	0.102*	0.60
H19B	0.3247	0.6408	0.0288	0.102*	0.60
C20	0.29543 (14)	0.3442 (11)	0.0017 (4)	0.090 (2)	0.60
H20A	0.2823	0.3655	−0.0546	0.135*	0.60
H20B	0.2809	0.3966	0.0292	0.135*	0.60
H20C	0.3009	0.1841	0.0128	0.135*	0.60
C19'	0.3332 (3)	0.440 (2)	0.0253 (2)	0.0852 (16)	0.40
H19C	0.3387	0.3178	−0.0040	0.102*	0.40
H19D	0.3409	0.5849	0.0106	0.102*	0.40
C20'	0.2936 (2)	0.446 (2)	0.0062 (7)	0.090 (2)	0.40
H20D	0.2805	0.4722	−0.0499	0.135*	0.40
H20E	0.2885	0.5680	0.0356	0.135*	0.40
H20F	0.2863	0.3021	0.0206	0.135*	0.40
C21	0.44184 (6)	0.2255 (4)	0.21482 (11)	0.0509 (5)	
H21A	0.4379	0.2886	0.1635	0.076*	
H21B	0.4257	0.0984	0.2083	0.076*	
H21C	0.4665	0.1736	0.2404	0.076*	

### Atomic displacement parameters (Å^2^)

**Table:**

	*U*^11^	*U*^22^	*U*^33^	*U*^12^	*U*^13^	*U*^23^
O1	0.0424 (7)	0.0596 (8)	0.0284 (6)	−0.0073 (6)	0.0136 (5)	0.0000 (5)
O2	0.0842 (11)	0.0693 (10)	0.0328 (7)	0.0258 (8)	0.0268 (7)	0.0055 (7)
O3	0.0640 (9)	0.0523 (8)	0.0420 (7)	0.0161 (7)	0.0223 (7)	0.0043 (6)
O4	0.0747 (11)	0.0811 (11)	0.0362 (7)	−0.0125 (9)	0.0197 (7)	0.0125 (7)
O5	0.0593 (10)	0.0871 (11)	0.0297 (7)	−0.0186 (8)	0.0051 (6)	−0.0025 (7)
O6	0.0460 (8)	0.0852 (10)	0.0295 (6)	0.0036 (7)	0.0118 (6)	−0.0072 (7)
N1	0.0523 (10)	0.0588 (10)	0.0246 (7)	0.0163 (8)	0.0140 (7)	0.0048 (7)
C1	0.0379 (10)	0.0466 (10)	0.0266 (8)	0.0022 (8)	0.0122 (7)	0.0019 (7)
C2	0.0402 (10)	0.0437 (9)	0.0268 (7)	−0.0006 (8)	0.0114 (7)	0.0038 (7)
C3	0.0416 (10)	0.0462 (10)	0.0258 (8)	−0.0044 (8)	0.0115 (7)	0.0019 (7)
C4	0.0437 (11)	0.0481 (10)	0.0263 (8)	−0.0017 (8)	0.0152 (7)	−0.0005 (7)
C5	0.0440 (11)	0.0482 (10)	0.0278 (8)	0.0061 (8)	0.0146 (7)	0.0024 (7)
C6	0.0378 (10)	0.0476 (10)	0.0242 (7)	−0.0014 (8)	0.0115 (7)	−0.0007 (7)
C7	0.0525 (12)	0.0580 (12)	0.0284 (8)	0.0146 (9)	0.0138 (8)	0.0051 (8)
C8	0.0570 (13)	0.0608 (12)	0.0293 (9)	0.0069 (10)	0.0124 (8)	0.0079 (8)
C9	0.0430 (11)	0.0497 (10)	0.0329 (8)	0.0041 (9)	0.0136 (8)	0.0002 (8)
C10	0.0666 (15)	0.0548 (12)	0.0534 (12)	0.0160 (11)	0.0264 (11)	0.0000 (10)
C11	0.0705 (17)	0.0824 (17)	0.0780 (16)	0.0095 (14)	0.0355 (14)	−0.0034 (14)
C12	0.0402 (11)	0.0537 (11)	0.0306 (8)	0.0018 (9)	0.0106 (7)	0.0046 (8)
C13	0.0475 (13)	0.0747 (15)	0.0566 (12)	0.0020 (11)	0.0193 (10)	0.0196 (11)
C14	0.0495 (15)	0.109 (2)	0.0837 (18)	−0.0089 (15)	0.0269 (13)	0.0163 (16)
C15	0.0408 (14)	0.121 (2)	0.0760 (17)	0.0139 (15)	0.0087 (12)	0.0119 (17)
C16	0.0581 (16)	0.0904 (19)	0.0672 (15)	0.0222 (14)	0.0079 (12)	0.0212 (14)
C17	0.0514 (13)	0.0600 (13)	0.0484 (11)	0.0060 (10)	0.0111 (10)	0.0112 (10)
C18	0.0442 (11)	0.0573 (11)	0.0304 (8)	−0.0019 (9)	0.0122 (8)	0.0004 (8)
C19	0.066 (2)	0.145 (3)	0.0269 (11)	−0.014 (2)	0.0000 (12)	0.0007 (15)
C20	0.066 (2)	0.125 (7)	0.0596 (19)	0.000 (3)	0.0030 (16)	0.003 (4)
C19'	0.066 (2)	0.145 (3)	0.0269 (11)	−0.014 (2)	0.0000 (12)	0.0007 (15)
C20'	0.066 (2)	0.125 (7)	0.0596 (19)	0.000 (3)	0.0030 (16)	0.003 (4)
C21	0.0642 (14)	0.0573 (12)	0.0362 (9)	0.0049 (10)	0.0253 (9)	−0.0034 (9)

### Geometric parameters (Å, °)

**Table:**

O1—C4	1.437 (2)	C10—C11	1.506 (3)
O1—H1O	0.9360	C10—H10A	0.9900
O2—C9	1.229 (2)	C10—H10B	0.9900
O3—C9	1.356 (2)	C11—H11A	0.9800
O3—C10	1.447 (2)	C11—H11B	0.9800
O4—C18	1.202 (2)	C11—H11C	0.9800
O5—C18	1.343 (2)	C12—C13	1.384 (3)
O5—C19'	1.456 (3)	C12—C17	1.393 (3)
O5—C19	1.470 (3)	C13—C14	1.380 (3)
O6—C8	1.416 (2)	C13—H13	0.9500
O6—H6O	0.9543	C14—C15	1.376 (4)
N1—C6	1.358 (2)	C14—H14	0.9500
N1—C7	1.444 (2)	C15—C16	1.370 (4)
N1—H1N	0.9087	C15—H15	0.9500
C1—C6	1.380 (3)	C16—C17	1.381 (3)
C1—C9	1.445 (2)	C16—H16	0.9500
C1—C2	1.534 (2)	C17—H17	0.9500
C2—C12	1.520 (3)	C19—C20	1.515 (3)
C2—C3	1.550 (2)	C19—H19A	0.9900
C2—H2	1.0000	C19—H19B	0.9900
C3—C18	1.508 (2)	C20—H20A	0.9800
C3—C4	1.528 (3)	C20—H20B	0.9800
C3—H3	1.0000	C20—H20C	0.9800
C4—C21	1.531 (2)	C19'—C20'	1.523 (3)
C4—C5	1.535 (2)	C19'—H19C	0.9900
C5—C6	1.512 (2)	C19'—H19D	0.9900
C5—H5A	0.9900	C20'—H20D	0.9800
C5—H5B	0.9900	C20'—H20E	0.9800
C7—C8	1.519 (2)	C20'—H20F	0.9800
C7—H7A	0.9900	C21—H21A	0.9800
C7—H7B	0.9900	C21—H21B	0.9800
C8—H8A	0.9900	C21—H21C	0.9800
C8—H8B	0.9900		
			
C4—O1—H1O	110.6	C11—C10—H10A	109.9
C9—O3—C10	117.92 (15)	O3—C10—H10B	109.9
C18—O5—C19'	118.3 (3)	C11—C10—H10B	109.9
C18—O5—C19	115.9 (2)	H10A—C10—H10B	108.3
C19'—O5—C19	11.3 (10)	C10—C11—H11A	109.5
C8—O6—H6O	107.7	C10—C11—H11B	109.5
C6—N1—C7	126.98 (15)	H11A—C11—H11B	109.5
C6—N1—H1N	113.8	C10—C11—H11C	109.5
C7—N1—H1N	119.0	H11A—C11—H11C	109.5
C6—C1—C9	119.37 (15)	H11B—C11—H11C	109.5
C6—C1—C2	121.94 (15)	C13—C12—C17	117.61 (19)
C9—C1—C2	118.67 (15)	C13—C12—C2	121.15 (17)
C12—C2—C1	114.57 (14)	C17—C12—C2	121.05 (17)
C12—C2—C3	107.33 (14)	C14—C13—C12	121.2 (2)
C1—C2—C3	110.10 (14)	C14—C13—H13	119.4
C12—C2—H2	108.2	C12—C13—H13	119.4
C1—C2—H2	108.2	C15—C14—C13	120.3 (2)
C3—C2—H2	108.2	C15—C14—H14	119.9
C18—C3—C4	113.02 (14)	C13—C14—H14	119.9
C18—C3—C2	108.21 (15)	C16—C15—C14	119.5 (2)
C4—C3—C2	111.72 (14)	C16—C15—H15	120.2
C18—C3—H3	107.9	C14—C15—H15	120.2
C4—C3—H3	107.9	C15—C16—C17	120.4 (2)
C2—C3—H3	107.9	C15—C16—H16	119.8
O1—C4—C3	110.45 (14)	C17—C16—H16	119.8
O1—C4—C21	110.28 (14)	C16—C17—C12	121.0 (2)
C3—C4—C21	112.11 (15)	C16—C17—H17	119.5
O1—C4—C5	106.80 (14)	C12—C17—H17	119.5
C3—C4—C5	107.19 (13)	O4—C18—O5	123.21 (15)
C21—C4—C5	109.82 (15)	O4—C18—C3	125.72 (16)
C6—C5—C4	115.09 (15)	O5—C18—C3	111.03 (16)
C6—C5—H5A	108.5	O5—C19—C20	107.3 (3)
C4—C5—H5A	108.5	O5—C19—H19A	110.3
C6—C5—H5B	108.5	C20—C19—H19A	110.3
C4—C5—H5B	108.5	O5—C19—H19B	110.3
H5A—C5—H5B	107.5	C20—C19—H19B	110.3
N1—C6—C1	122.83 (15)	H19A—C19—H19B	108.5
N1—C6—C5	115.03 (15)	O5—C19'—C20'	107.8 (3)
C1—C6—C5	122.12 (14)	O5—C19'—H19C	110.1
N1—C7—C8	109.65 (15)	C20'—C19'—H19C	110.1
N1—C7—H7A	109.7	O5—C19'—H19D	110.1
C8—C7—H7A	109.7	C20'—C19'—H19D	110.1
N1—C7—H7B	109.7	H19C—C19'—H19D	108.5
C8—C7—H7B	109.7	C19'—C20'—H20D	109.5
H7A—C7—H7B	108.2	C19'—C20'—H20E	109.5
O6—C8—C7	112.12 (17)	H20D—C20'—H20E	109.5
O6—C8—H8A	109.2	C19'—C20'—H20F	109.5
C7—C8—H8A	109.2	H20D—C20'—H20F	109.5
O6—C8—H8B	109.2	H20E—C20'—H20F	109.5
C7—C8—H8B	109.2	C4—C21—H21A	109.5
H8A—C8—H8B	107.9	C4—C21—H21B	109.5
O2—C9—O3	120.81 (16)	H21A—C21—H21B	109.5
O2—C9—C1	126.18 (17)	C4—C21—H21C	109.5
O3—C9—C1	112.98 (15)	H21A—C21—H21C	109.5
O3—C10—C11	109.02 (18)	H21B—C21—H21C	109.5
O3—C10—H10A	109.9		
			
C6—C1—C2—C12	134.86 (18)	C6—C1—C9—O2	−14.7 (3)
C9—C1—C2—C12	−46.3 (2)	C2—C1—C9—O2	166.41 (19)
C6—C1—C2—C3	13.8 (2)	C6—C1—C9—O3	163.31 (16)
C9—C1—C2—C3	−167.34 (16)	C2—C1—C9—O3	−15.6 (2)
C12—C2—C3—C18	59.04 (18)	C9—O3—C10—C11	−102.7 (2)
C1—C2—C3—C18	−175.64 (14)	C1—C2—C12—C13	−52.1 (2)
C12—C2—C3—C4	−175.91 (13)	C3—C2—C12—C13	70.5 (2)
C1—C2—C3—C4	−50.59 (18)	C1—C2—C12—C17	133.07 (19)
C18—C3—C4—O1	71.70 (18)	C3—C2—C12—C17	−104.3 (2)
C2—C3—C4—O1	−50.64 (17)	C17—C12—C13—C14	−0.4 (3)
C18—C3—C4—C21	−51.7 (2)	C2—C12—C13—C14	−175.5 (2)
C2—C3—C4—C21	−174.06 (14)	C12—C13—C14—C15	0.0 (4)
C18—C3—C4—C5	−172.31 (15)	C13—C14—C15—C16	−0.1 (4)
C2—C3—C4—C5	65.35 (18)	C14—C15—C16—C17	0.6 (4)
O1—C4—C5—C6	75.18 (19)	C15—C16—C17—C12	−1.0 (4)
C3—C4—C5—C6	−43.2 (2)	C13—C12—C17—C16	0.9 (3)
C21—C4—C5—C6	−165.23 (16)	C2—C12—C17—C16	175.9 (2)
C7—N1—C6—C1	178.34 (19)	C19'—O5—C18—O4	1.5 (8)
C7—N1—C6—C5	−2.8 (3)	C19—O5—C18—O4	−11.0 (5)
C9—C1—C6—N1	6.8 (3)	C19'—O5—C18—C3	179.3 (7)
C2—C1—C6—N1	−174.32 (17)	C19—O5—C18—C3	166.8 (5)
C9—C1—C6—C5	−171.97 (17)	C4—C3—C18—O4	−58.8 (3)
C2—C1—C6—C5	6.9 (3)	C2—C3—C18—O4	65.5 (3)
C4—C5—C6—N1	−170.10 (16)	C4—C3—C18—O5	123.50 (18)
C4—C5—C6—C1	8.8 (3)	C2—C3—C18—O5	−112.22 (18)
C6—N1—C7—C8	−156.00 (19)	C18—O5—C19—C20	−167.3 (4)
N1—C7—C8—O6	−58.2 (2)	C19'—O5—C19—C20	88 (2)
C10—O3—C9—O2	−15.2 (3)	C18—O5—C19'—C20'	−133.8 (6)
C10—O3—C9—C1	166.70 (17)	C19—O5—C19'—C20'	−53 (2)

### Hydrogen-bond geometry (Å, °)

**Table:**

*D*—H···*A*	*D*—H	H···*A*	*D*···*A*	*D*—H···*A*
O1—H1O···O6^i^	0.94	1.92	2.802 (2)	157
O1—H1O···O4	0.94	2.61	3.061 (2)	110
O6—H6O···O1^ii^	0.95	1.78	2.727 (2)	172
N1—H1N···O2	0.91	1.91	2.650 (2)	137

Symmetry codes: (i) *x*, −*y*+1, *z*−1/2;  (ii) −*x*+1, −*y*+1, −*z*+1.
